# The United States Health Care System is Sick: From Adam Smith to Overspecialization

**DOI:** 10.7759/cureus.2720

**Published:** 2018-05-31

**Authors:** Deanna Anderlini

**Affiliations:** 1 HMNS, The University of Queensland

**Keywords:** healthcare system, family doctor, healthcare cost, overspecialization, nih

## Abstract

The United States (US) health care system is sick. High cost affects the nation and the people. The poor outcomes mainly impact the patients. If we do not fix it, the system will implode because of the unsustainable economic burden.

The choice to shape the health care system on the “pin factory” model described by Adam Smith is the cause of the failure. The key players in the health care system are accordingly molded.

Many factors tightly intertwined need to be addressed. Medical education, the role of family doctor, the overspecialization and, not least, the translation of discoveries into clinical practice are among them.

The failure of the US health care system is a complex and multi-factorial issue which requires a global approach.

## Introduction and background

Health and equality are inextricably linked. It has been that way in each society, from ancient time till today.

If we think of the health care system as something new, we are wrong. Written records and physical remains found in Deir el-Medina in Egypt reveal the first documented governmental health care system running around 1292 B.C. Workers were entitled to paid sick days and to free check-ups at clinics. Not less astonishing is the excavation of a hospital in the Angkor Wat archaeological site [1]. At the beginning of the 19th century, a desire to expand medical knowledge arose as the consequence of technological advances (thermometer, stethoscope, vaccine, anesthesia and many more). This encouraged clinical researchers to further specialize because they thought that only specialization would enable them to acquire deeper knowledge. Moreover, the administrative rationality constraints, imposed by the need to manage larger populations, have prompted the gathering of individuals with the same disease and, as a consequence, the emergence of subclinical [2]. The practice of Medicine shifted from a pre-scientific holistic approach to the modern perspective supported by scientific explanations of pathology. Foucault put the turning point at the reorganization of hospitals and epidemics of the 18th century [3].

The definition of what means to be healthy has evolved many times since the early days of mankind reflecting beliefs and medical knowledge [4]. The journey from medical Egyptian papyri to the rod of Asclepius, from Hippocrates to Galen, brought us to the 1948 World Health Organisation (WHO) definition of health as “a state of complete physical, mental and social well-being, not merely the absence of infirmity or disease”. Later the International Conference on Primary Health Care released the Alma-Ata Declaration. It is a milestone in the field of public health: primary health care was identified as the key to reach health for All.

Unfortunately, our 21st-century health care system mirrors the 200 years old model of factory described by Adam Smith with specialists playing the role of pin-makers [5]. The issue is that we can split a job into smaller parts, but we cannot split the patient into smaller parts without losing the whole view. Fordism applied to health care results in patients becoming bodies made of parts/organs in need of maintenance/service/cure which in turn require factories/clinics. We can see a high resemblance between the factory system masterfully depicted by Chaplin in “Modern Times” and our “Modern Hospitals” filled by medical sub-sub-specializations.

Everything down the path is thought accordingly to that system. We have research trials focus on patients with only one disease to avoid confounding variables (and missing the reality). We see specialists that rarely know how their specific therapies interact with others’. Redundant tests due to lack of communication and discontinuity in the care are the daily routine. Everything, from the medical education to the training, from funding and planning, is compartmentalized in a vicious circle [6-8].

Even more of a concern is that health care treated as a commodity in a market arranged to create value for the provider rather than the patient. As a consequence, the main goal shifts from the wellbeing of people to productivity and making money. The Fordism, well described by Gramsci in his 1934 essay [9], pervades each and every aspect of the system. Hospitals are built with units looking like the assembly line model of production. The sub-division of medical tasks is the norm [10].

## Review

First and foremost, we have to acknowledge that health care is a multi-factorial service. The only way we can improve it is anything but a global approach, very far from an overspecialized fragmented intervention.

Second, the huge cost related to the health care would make you think that it is strictly dependent on economics, but in reality the major player is politics as “the” political institution decides how to spend the budget: health care is political [11,12].

In this context, even the Affordable Care Act (ACA) becomes not compatible with the Hippocratic ethos of caring and curing because no matter if its goal is a good, affordable care for all Americans, health is now a big business where fee-for-service is the rule: “unfortunately, the goals of the ACA is primarily bureaucratic and financial” [13] .

We will take into consideration the main points related to health care by defining the problem and our solution.

Health care system

Even conservative estimates suggest that nearly a quarter of US patients suffer multi-morbidity, a figure reiterated in the majority of the countries members of the Organization for Economic Cooperation and Development (OECD). Multi-morbidity is the norm rather than the exception. By the age of 65, almost all people present with two or more diseases, and in developing countries this is true from as early as the age of 55 [14]. The Robert Wood Johnson Foundation report shows how 75 million Americans with multi-morbidity which represents 25% of the population account for 65% of health expenditure [15, 16]. Once more the 2008 WHO report singled out the fragmentation of care as one of the five shortcomings in the way health care is delivered.

A patient is not a patchwork of single symptoms as the physiology of the human body is not a collection of independent modules: he is a complex interacting whole who requires a global approach [7]. For instance, the outcomes of the fragmented health care system in the US accounted for 100 million medication errors per year as revealed by a study in 2000.

A total of 250,000 or more unnecessary deaths per year due to errors were reported in 2016 [17]. The U.S. spent about $7,848 per capita in 2008 on health care with specialists as the biggest drivers of the cost. In 2017 the cost rose to 10,833 per capita [18]. Similar results come from the UK where the latest study, published in 2018, gave an account of more than 200 million medication errors [19]. The figures are staggering, especially if we compare the cost with poorer outcomes [20].

New models have been tried with good results. A new model of health care system was established in Appleton: the aim was to deliver a better value to the patient. A group of 16 forward-thinking organizations in North America formed the Health Care Value Leaders Network based on this model. They looked at every step in the process of care and found out that over 80% of all steps do not provide any value. By removing those unnecessary steps, the Network improved quality and reduced the costs. Visits to the ER decreased by 29%, hospital admissions by 11% [21].

In 2001, the Thailand Universal Coverage Health Insurance started a new system based on a capitation basis: each citizen, either healthy or ill, not covered by insurance must register with a hospital which will get $30 flat rate/year/capita. This way hospital in overpopulated areas, which could not afford to pay staff because located in poor regions, will have enough money to hire more doctors. As a result of the reform, many more citizens will get access to treatments [22].

The University of Michigan with its project has been able to make huge savings [23]. The Transforming care at bedside achieved 30% drop in the cost of inpatient care and Gundersen Luthera’s end-of-life care process is 50% less expensive than the national average per Medicare enrollee.

The Meikirch Model conceived in a small village in Switzerland in 2014 began with a new, broader concept of health where biological given potential (BGP) and personally acquired potential (PAP) are taken into consideration [24]. Accordingly, with the new concept, health value is measured on the outcomes and, financially revolutionary, payment is done neither for-performance nor for value, just for health care based on BGP and PAP.

Harvard economist, Michael Porter suggested a value-based system [7] where outcomes (including readmission and mortality) are measured and compared. The model has been implemented at the University of Utah Health Care since 2012: the results showed a -15% cost/year and better outcomes. The "opportunity index” which sets initial priorities was instrumental in lowering the costs.

Medical education and the role of university

The two important events that shaped medical education in the US are as follows:

1. The Flexner report published in 1910 suggested higher admission standard for medical schools and to deliver mainly science in teaching and research [25]. Flexner himself thought that the medical education in the US based on his report had moved too much towards science.

2. The birth of schools of public health in 1916 supported by the Rockefeller Foundation. It generated a schism between Medicine and public health. Earlier, the Pasteur Institute in Paris (1888) and the Koch Institute in Berlin (1891) had been established, but they did not have a major role in the medical education. Nowadays the John Hopkins Bloomberg School of Public Health has 1638 staff, a budget of $529 million and it is one of the top recipients of NIH grants [26].

The outcome is a fragmented, one-sided education, too narrow for a global view of patients and too rigid to adapt to changing needs of the aging population. The doctor/patient relationship is a very powerful instrument; critics of super specialization which can lead to a distorted relationship has been pointed out by philosophers as Habermas [27,28].

Interdisciplinary and cross-field courses will not solve the problem of the too narrow basic education because they occur at an already highly specialized level. What is needed more is a school delivering a basic general knowledge to everybody and enabling wide communication among students. Specialized professional schools provide a background too narrow, and they predetermine the path of the student at a very early stage, making the exchange of ideas impossible but with people in the same field.

We have to broaden the basic knowledge starting at the undergraduate level because many courses are already too narrow and overspecialized creating young professionals without the flexibility necessary to the continuous changing circumstances and with gaps which will never be filled.

Post-graduate training needs a wider perspective, with biology instead of molecular biology, physiology for scientists, research and analysis methods for doctors [29]. The quota for training specialists should be controlled to curb the number of specialists and to avoid losing general physicians, a phenomenon occurring not just in the US, but in countries like India and the UK which experience the shortage of general internists [30,31].

There is a push for a “remoralisation” of health professionals’ education contraposed to the entrenched collusion to preserve their influence. Medical education has to be entrenched with values and practice of social justice and equity in health care. Health professionals should also mobilize knowledge. Many important skills – as leadership, management, communication – are neglected in the medical curriculum and this confirms how universities are little appreciated as a core social institution [32].

Overspecialization

The first US specialty board (Ophthalmology) was established in 1917 followed by dozen other specialties [33]. Currently, there are over 120 medical specialties and sub-specialties. From 1940, when there was little specialization, to 1975, medical expenses increased from $3 billion to $75 billion. Health care expenditure now constitutes 1/6 of the GDP in the US [34]. In 2012, Forbes published an article titled: “Why are U.S. health care cost so high?” the answer was: specialists with higher per-procedure rates paid by both private and public payers [35]. Many other investigations came to the same conclusion: we have too few primary-care physicians and too many specialists.

Overspecialization in health care has been a failure for patients and budget altogether [36]. Prof. Cueto remarked how essential was to eradicate medical overspecialization. In his view, the top-down health campaigns had to move towards a community participation model [37]. Specialization is inevitable and useful. The sub-sub-specializations are the real danger. The unbalance between the number of specialists and generalists is the threat. Furthermore, overspecialization favors one-sidedness and it prevents the grasp of a more general global knowledge.

General physician, family doctor

In the marvelous portrait by Balzac, we see the Country Doctor listening to and taking care of the patient in each stage of his life: from birth to death (which is the natural termination of a physical malady) and funeral included. Now that role has left room to the scientific presence of the clinician who makes a diagnosis and prescribes pills excluding the death as not as part of his duty anymore [38]. The family doctor slowly lost his importance in the health system, more and more under pressure and struggling to bring his help as described by Kafka and Muir [39]; his satisfaction is now mainly related within the community than to earnings.

The “golden age of Medicine” spanning from 1946 to 1970 is long gone. The time when doctors enjoyed the monopoly of knowledge and great power in society has been superseded by an era where physicians feel cognitive dissonance due to the mismatch between their idea of medicine, as honorable art, and the production lines they now belong to. Academization of other health professions as nurses, radiologists and psychologists has eroded their field of action. The lack of support from the State who “finally caught up with the last guild” makes this profession less attractive to young students.

Despite the fact that general physicians (GPs) are the healthiest segment of populations, they are stressed and burned out, overworked, underpaid, under time pressure to check up patients in less than 10 minutes [40]. Physicians have to rely more on referrals with rates of referrals doubling in the past decade. Hospitals, organized in super-specialized departments, are also forced to hire highly paid specialists. Last, the lack of professional reward and the wrong diffused social disapproval of the role of GP, explain the decline of the number of students choosing Family Medicine in the last five years, which, in turns, will produce a shortage of the most needed figure in the future [41].

Keeping in mind that family physicians are the first contact for health problems and they directly address most of the health care needs, they are ideal leaders of the system as stated in 2nd Future of Family Medicine project [42]. If we want to improve the care for patients, we have to give back the GP his dignity in society. To do so we have to scratch the compensation model, on which the health care system is based now, which rewards piecework, procedures and technology to establish a new system where general physician finally can sit and listen to his patient: time is essential for it.

The Meltzer project was funded under the ACA, and the implementation began in 2012. It describes a model where a single physician provides in- and outpatient care for each patient; this way it enables the necessary global view of the patient and the continuity of care. The workload of the physician, now close to 2000 patients/year, would be 200, giving him time to think and to rebuild the broken doctor/patient relationship [43]. In his vision, the primary care physician will split his day between the hospital and the outpatients to promote collaboration and spreading of knowledge [44]. We believe that the main feature to become a good doctor is “interest in humanity” because he only will be able to see the patient as an individual and not as a number.

Translating findings into clinical practice (Valley of Death)

Health is considered a basic right for any human being. When millions of individuals have no access to health care, it goes against human rights’ ethic to financially support medical research if it is not translatable into clinical practice [45]. Time, minds, money and lives cannot be wasted while a few pursue glory and gratification.

Barriers between scientists and clinicians are rooted first in a decade of specialized education through two languages, so at the end they cannot communicate. Second, they lack a common value system: views, questions and rewards are different. Last, the sources of passion and intensity of emotions do not match: doctors deal with life and death, scientists don’t. These barriers jeopardize the translation of discoveries into practice.

The decision by Pharma giant Pfizer to pull out of research into Alzheimer's is a painful lesson [46]. The justification for that choice was that “more than 99% of trials for Alzheimer's drugs have failed in the past 15 years”; this mainly happens at phase III. All this is a clear sign that basic-fragmented research is too costly to be feasible any longer. Probably others private research institutions will follow because a positive financial result is at their core. Not less important is the waste of tax-payers money.

Research/funding

Research plays an essential role in improving health care. It requires good minds, time and money. Sadly enough, medical research has never been such a huge pressure from outside forces trying to dictate the agenda [47]. For too long, scientific productivity of researchers has been measured by the H-Index which depends on the number of papers and citations, but no correlation with the quality of the publications. A new model of funding should be based on the replicability and significance of the protocol; it should reward the rate of translation into practice and the outcomes achieved by the applicant researcher [48].

Offers of cross-field fellowships and transdisciplinary research awards would be also good incentives to break compartmentalization and to favor future collaborations.

The NIH funding criteria need more than the National Centre for Advancing Translational Sciences announced in 2011 to give clinical research a new life. We believe that seven key points need to be addressed:

1. Financial support for medical students who accumulate $180,000 debt to get graduated.

2. Incentives for physician-scientists who face a longer training period, compared to biomedical researchers.

3. Greater funding for clinicians-scientist.

4. Grants exclusively for translational science.

5. Renewability of funds based on the obtained rate of translated results into practice.

6. Rotation every five years for a clinician to be the principal investigator in basic research and for a biomedical researcher to guide a clinical trial.

7. Not least, the peer-review process must balance its favor equally between hypothesis-driven research and applied research. Only an NIH peer-review panel with equal numbers of MDs and PhDs would reach that goal.

Medical publications

Time has confirmed that a publication in itself does not guarantee the dissemination of results and translation into clinical practice, hence the translation becomes primarily the researcher’s responsibility. Arturo Casadevall pointed out that the way scientists create specialized groups is highly similar to the guild system of the Middle Ages. Moreover, overspecialization is conducive of judging scientific work by the impact factor of the journal in which the paper is published because scientists become less able to evaluate works outside their field of expertise.

Scientists should publish less quantity and more quality papers [49]. A maximum of one paper/year should be set to give them time to improve the quality.

Publishers, not to forget, are private enterprises listed on share markets [50] and born to create profits. Nonetheless, they have become an integral part of the medical world. They too should swear to uphold the Hippocratic Oath: “first, do no harm.”

## Conclusions

What we need most is the will at the leadership level in each sector to do it, from medical education to research, from funding to planning, not least from publishing journals. Scientific journals are the natural places for debates and meeting points of different fields. They can be the forces promoting a new way of thinking.
